# Controlled Synthesis and Crystallization-Driven Self-Assembly of Poly(*ε*-caprolactone)-*b*-polysarcosine Block Copolymers

**DOI:** 10.3390/molecules30153108

**Published:** 2025-07-24

**Authors:** Zi-Xian Li, Chen Yang, Lei Guo, Jun Ling, Jun-Ting Xu

**Affiliations:** 1 State Key Laboratory of Biobased Transportation Fuel Technology, Department of Polymer Science and Engineering, Zhejiang University, Hangzhou 310058, China; 3150101189@zju.edu.cn (Z.-X.L.); 11929024@zju.edu.cn (C.Y.); 3170100816@zju.edu.cn (L.G.); 2 MOE Key Laboratory of Macromolecular Synthesis and Functionalization, Department of Polymer Science and Engineering, Zhejiang University, Hangzhou 310058, China

**Keywords:** amphiphilic block copolymers, semicrystalline block copolymers, crystallization-driven self-assembly, poly(*ε*-caprolactone), polysarcosine, self-seeding, lamellar crystals

## Abstract

Poly(ε-caprolactone)-*b*-polysarcosine (PCL-*b*-PSar) block copolymers (BCPs) emerge as a promising alternative to conventional poly(*ε*-caprolactone)-*b*-poly(ethylene oxide) BCPs for biomedical applications, leveraging superior biocompatibility and biodegradability. In this study, we synthesized two series of PCL-*b*-PSar BCPs with controlled polymerization degrees (*DP* of PCL: 45/67; *DP* of PSar: 28–99) and low polydispersity indexes (*Đ* ≤ 1.1) and systematically investigated their crystallization-driven self-assembly (CDSA) in alcohol solvents (ethanol, *n*-butanol, and *n*-hexanol). It was found that the limited solubility of PSar in alcohols resulted in competition between micellization and crystallization during self-assembly of PCL-*b*-PSar, and thus coexistence of lamellae and spherical micelles. To overcome this morphological heterogeneity, we developed a modified self-seeding method by employing a two-step crystallization strategy (i.e., *T*_c1_ = 33 °C and *T*_c2_ = 8 °C), achieving conversion of micelles into crystals and yielding uniform self-assembled structures. PCL-*b*-PSar BCPs with short PSar blocks tended to form well-defined two-dimensional lamellar crystals, while those with long PSar blocks induced formation of hierarchical structures in the PCL_45_ series and polymer aggregation on crystal surfaces in the PCL_67_ series. Solvent quality notably influenced the self-assembly pathways of PCL_45_-*b*-PSar_28_. Lamellar crystals were formed in ethanol and *n*-butanol, but micrometer-scale dendritic aggregates were generated in *n*-hexanol, primarily due to a significant Hansen solubility parameter mismatch. This study elucidated the CDSA mechanism of PCL-*b*-PSar in alcohols, enabling precise structural control for biomedical applications.

## 1. Introduction

Amphiphilic block copolymers (BCPs), comprising covalently bonded polymer blocks with distinct hydrophilicity and hydrophobicity, form various self-assembled structures in selective solvents such as spheres, cylinders, lamellae, vesicles, and multidimensional hierarchical assemblies [1,2,3,4]. These morphological diversity and size tunability confer unique advantages on amphiphilic BCPs as building blocks for functional microstructured materials across diverse fields [5,6]. While the self-assembly of amorphous BCPs is governed by the differential solubility of the constituent blocks [2], in semicrystalline BCPs, it is determined by the interplay between micellization and crystallization [7,8]. For semicrystalline BCPs, crystallization-driven self-assembly (CDSA) provides a robust approach for preparing non-spherical self-assembled structures with precise control over structural uniformity. These anisotropic structures offer significant benefits in fields such as nanoelectronics, composite reinforcement, and therapeutic agent delivery [6,9]. Extensive studies focus on CDSA of BCPs containing crystalline core-forming blocks, including poly(ferrocenyldimethylsilane) (PFS) [10,11,12,13,14], poly(ethylene oxide) (PEO) [15,16,17], polyethylene (PE) [18,19,20,21], poly(L-lactic acid) (PLLA) [22,23,24,25], poly(*ε*-caprolactone) (PCL) [26,27,28,29,30,31,32], polypeptoids [33,34,35], and others [6,36,37,38,39,40].

PCL-*b*-PEO is a typical semicrystalline BCP in which the PCL block exhibits excellent biocompatibility and biodegradability [41], while the PEO block is hydrophilic, protein-resistant, and bioinert [42]. These combined properties drive its biomedical applications and CDSA research [17,43,44,45,46,47,48]. However, emerging concerns over the chronic toxicity and unanticipated immunogenicity of PEO [42,49,50,51] necessitate alternatives. Polysarcosine (PSar) is an amino acid-based synthetic polymer whose structural unit is the dehydration residue of the endogenous amino acid sarcosine. PSar not only demonstrates hydrophilicity and protein resistance comparable to PEO [52,53,54], but also exhibits improved biodegradability [54]. Accumulated studies have suggested that PSar possesses superior biocompatibility and lower immunogenicity compared with PEO [55,56,57,58]. These outstanding characteristics position PSar as a potential candidate for PEO, stimulating intensive research interest in the synthesis strategies and self-assembly behavior of PSar-containing copolymers [54,57,59,60,61,62,63].

Leveraging their biocompatibility, biodegradability, and amphiphilic structure, PCL-*b*-PSar BCPs hold promise for applications in drug delivery, theranostics, and tissue engineering. However, systematic investigations into their self-assembly behavior remain limited. Guo’s group initially synthesized PSar-*b*-PCL through sequential ring-opening polymerization (ROP) of sarcosine *N*-carboxyanhydride (NCA) and *ε*-caprolactone (*ε*-CL) [59], yielding ~100 nm aqueous spherical nanoparticles. In their subsequent study, star-shaped three-armed PSar-*b*-PCL BCPs were prepared [60]. With a fixed molecular weight of PCL, the size of the spherical aggregates in aqueous solution decreased as the weight fraction of PSar increased. Deng et al. synthesized PCL-*b*-PSar diblock and PSar-*b*-PCL-*b*-PSar triblock copolymers, investigating the thermoresponsive self-assembly behavior of the latter in aqueous solution [61]. Nanofibers and unilamellar sheets that initially formed at ambient conditions changed into worm-like cylinders and spheres upon thermal treatment at 65 °C. Rising to 90 °C further induced the formation of multilamellar polymersomes.

Although the aqueous self-assembly behavior of PCL-*b*-PSar BCPs has been documented [59,60,61], the observed size polydispersity and morphological heterogeneity suggest that the self-assembly pathways may be primarily governed by micellization rather than crystallization. To the best of our knowledge, no studies to date have reported the fabrication of morphologically uniform PCL-*b*-PSar assemblies using CDSA approaches. Furthermore, the interplay between micellization and crystallization governing the self-assembly pathway of these BCPs has not been investigated in detail. Herein, we successfully synthesized PCL-*b*-PSar BCPs with low polydispersity indexes (*Đ* ≤ 1.1) and systematically investigated their CDSA behavior in alcohol solvents, focusing on the effects of crystallization temperature, BCP composition, and solvent quality. Results revealed that the limited solubility of PSar in alcohols led to competition between micellization and crystallization during self-assembly, accounting for the observed morphological heterogeneity. To overcome this challenge, we modified the conventional self-seeding method by adopting a two-step crystallization strategy, in which amorphous micelles could be converted into crystals and uniform self-assembled structures were obtained.

## 2. Results and Discussion

### 2.1. Synthesis and Characterization of PCL-b-PSar

**Scheme 1:**
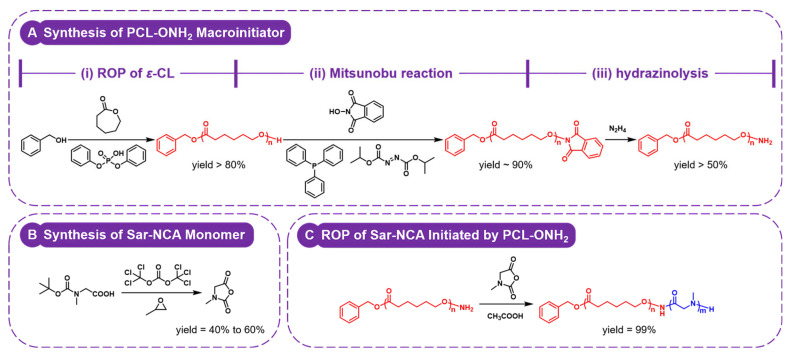
Synthesis route for PCL-*b*-PSar BCPs. (**A**) Synthesis of PCL–ONH_2_ macroinitiator: (**i**) ROP of *ε*-CL with benzyl alcohol/diphenyl phosphate; (**ii**) Mitsunobu reaction with *N*-hydroxyphthalimide; (**iii**) hydrazinolysis to expose the –ONH_2_ terminal group. (**B**) Synthesis of Sar-NCA monomer: Ring-closure of *N*-Boc-sarcosine with triphosgene. (**C**) ROP of Sar-NCA initiated by PCL–ONH_2_.

**Figure 1:**
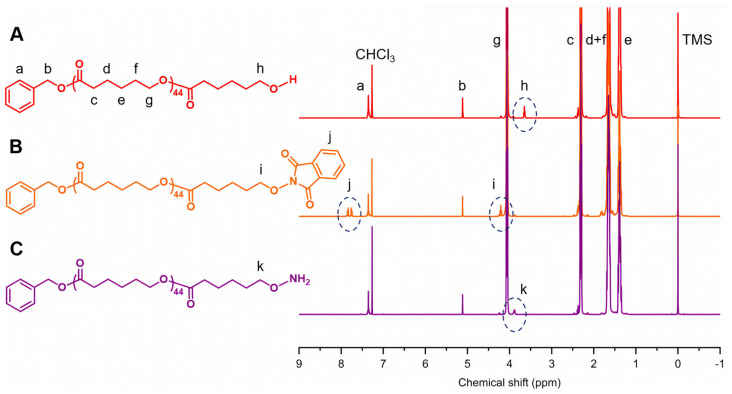
Typical ^1^H NMR spectra of (**A**) PCL, (**B**) PCL−O−NHP, and (**C**) PCL−ONH_2_ in CDCl_3_. The lowercase letters correspond to specific protons in the polymers with their respective signals in the spectra. Tetramethylsilane (TMS) was used as the internal standard.

The synthesis of PCL-*b*-PSar BCPs was inspired by the strategy originally proposed by Deng et al. [61], as illustrated in Scheme 1. In this study, two PCL homopolymers with controlled molecular weights and low polydispersity indexes (*Đ* ≤ 1.05) were synthesized via ROP of *ε*-CL using benzyl alcohol as the initiator and diphenyl phosphate as the acid organocatalyst [64], resulting in polymers bearing terminal hydroxyl groups. However, these terminal hydroxyl groups exhibit insufficient nucleophilicity to initiate the living ROP of NCA and *N*-thiocarboxyanhydride (NTA) monomers for synthesizing the PSar blocks [65,66]. To address this limitation, the hydroxyl groups were converted into more nucleophilic oxyamino groups through a two-step modification protocol involving Mitsunobu reaction followed by hydrazinolysis [67,68]. Figure 1 shows the typical ^1^H nuclear magnetic resonance (NMR) spectra of PCL, PCL−O−NHP, and PCL−ONH_2_. The number-average polymerization degrees (*DP*s) of the two PCL homopolymers are quantitatively determined from the integral ratio between the backbone methylene protons (*δ* = 2.14–2.46 ppm, –C*H*_2_COO–, peak c in Figure 1A) and the benzyl methylene protons (*δ* = 5.12 ppm, PhC*H*_2_–, peak b in Figure 1A) in ^1^H NMR spectra (with *DP* values of 45 and 67, respectively). The chemical shift of the terminal methylene protons from *δ* = 3.65 ppm (–C*H*_2_OH, peak h in Figure 1A) to *δ* = 4.21 ppm (–C*H*_2_–O–NHP, peak i in Figure 1B), along with the appearance of the aromatic proton signals at *δ* = 7.72–7.87 ppm (*N*-substituted phthalimide protons, peak j in Figure 1B), confirms the progress of the Mitsunobu reaction. Subsequently, the shift of the terminal methylene protons from *δ* = 4.21 ppm to *δ* = 3.89 ppm (–C*H*_2_ONH_2_, peak k in Figure 1C), accompanied by the disappearance of the aromatic signals, indicates the completion of the hydrazinolysis reaction. These characteristic spectral changes demonstrate the end-group transformation and the successful synthesis of oxyamino-terminated PCL macroinitiators.

**Figure 2:**
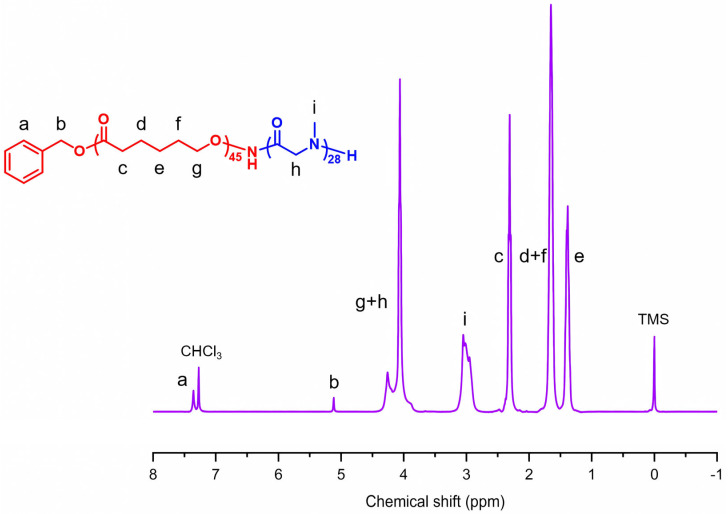
^1^H NMR spectrum of PCL_45_-*b*-PSar_28_ in CDCl_3_. The lowercase letters correspond to specific protons in the BCP with their respective signals in the spectrum. Tetramethylsilane (TMS) was used as the internal standard.

**Figure 3:**
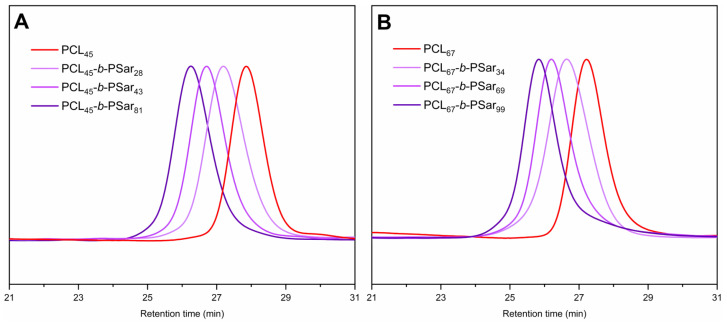
GPC traces of PCL homopolymers and PCL-*b*-PSar BCPs: (**A**) PCL_45_ and PCL_45_-*b*-PSar*_n_*, *n* = 28, 43, 81; (**B**) PCL_67_ and PCL_67_-*b*-PSar*_n_*, *n* = 34, 69, 99.

To prepare structurally controlled PCL-*b*-PSar BCPs, Sar-NCA was selected as the monomer for the synthesis of the PSar block. Sar-NCA was synthesized under ambient air and moisture-tolerant conditions [69]. 1,2-Epoxypropane was employed as an ultrafast scavenger of HCl to improve ring-closure efficiency and suppress acid-catalyzed decomposition. The effectiveness of this method is evidenced by the ^1^H NMR spectrum (Figure S1), which shows only signals corresponding to the product and residual solvents, with no impurity resonances detected. The ROP of Sar-NCA, initiated by PCL−ONH_2_ macroinitiators and catalyzed by acetic acid (AA) [70], afforded PCL-*b*-PSar BCPs with controlled PSar molecular weights. The ^1^H NMR spectrum of PCL-*b*-PSar is exemplarily shown in Figure 2. The *DP*s of PSar blocks are determined by the integral ratio between the methyl protons (*δ* = 2.75–3.25 ppm, −NCH_3_–, peak i in Figure 2) and the benzyl methylene protons (*δ* = 5.12 ppm, PhC*H*_2_–, peak b in Figure 2) in the ^1^H NMR spectra of PCL-*b*-PSar BCPs. All PCL homopolymers and BCPs exhibit narrow unimodal gel permeation chromatography (GPC) peaks, as shown in Figure 3. The number-average molecular weights (*M*_n_), polydispersity indexes, and weight ratios of the PCL homopolymers and the PCL-*b*-PSar BCPs investigated in this study are summarized in Table 1. For subsequent discussion, the polymers are designated based on *DP*s determined by ^1^H NMR results, such as PCL_45_-*b*-PSar_28_ (BCP) and PSar_51_ (homopolymer).

In comparison with Deng’s work [61], Sar-NTA ROP was replaced with Sar-NCA ROP in our synthesis, which offered two key improvements. First, the duration for monomer and BCP preparation was substantially reduced. Sar-NCA synthesis only requires less than 1 day with simplified purification. Nevertheless, synthesizing Sar-NTA demanded a multi-day process involving laborious chromatographic purification. Moreover, the ROP of Sar-NTA occurred at high temperature (60 °C) and lasted 1–2 days, while polymerization of Sar-NCA in this study could be conducted at room temperature and was finished within 30 min. More importantly, the structural control of the BCPs was improved. The lower reactivity of Sar-NTA hindered precise control over the *DP*s of PSar blocks via monomer-to-initiator ratios, and the high polymerization temperature induced side reactions, resulting in broadened molecular weight distributions (often > 1.2) with tailing or bimodality in GPC traces [61]. In contrast, Sar-NCA’s high reactivity ensured quantitative initiation and rapid room-temperature polymerization, enabling precise *DP* control solely through monomer-to-initiator ratios and yielding BCPs with low polydispersity indexes (*Đ* ≤ 1.1).

### 2.2. CDSA of PCL-b-PSar in Ethanol

In this study, we applied the well-established self-seeding method introduced by Blundell et al. [71] for preparing PCL-*b*-PSar crystalline micelles. From the morphological homogeneity perspective, the self-seeding method is applicable to the semicrystalline BCPs exhibiting a high critical micellization concentration (i.e., most of the BCPs exist in the form of unimers in the solution) and a slow nucleation rate at the isothermal crystallization temperature (*T*_c_). The preparation procedure is briefly described as follows. PCL-*b*-PSar is first dissolved in a selective solvent (specifically ethanol, *n*-butanol, or *n*-hexanol herein) at 70 °C, followed by quenching to room temperature for overnight crystallization. Subsequently, the suspension is maintained at the self-seeding temperature (*T*_s_) to dissolve most of the polymer crystals while preserving small crystalline nuclei. Finally, the solution is quenched to *T*_c_ for controlled crystal growth. The *T*_s_ was determined via turbidity measurements (Figure S2), which was identified as the temperature corresponding to the minimum UV-vis absorbance.

**Scheme 2:**
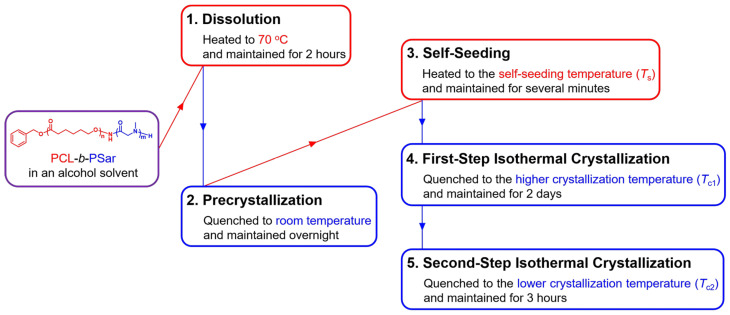
Self-seeding method coupled with two-step crystallization strategy.

**Figure 4:**
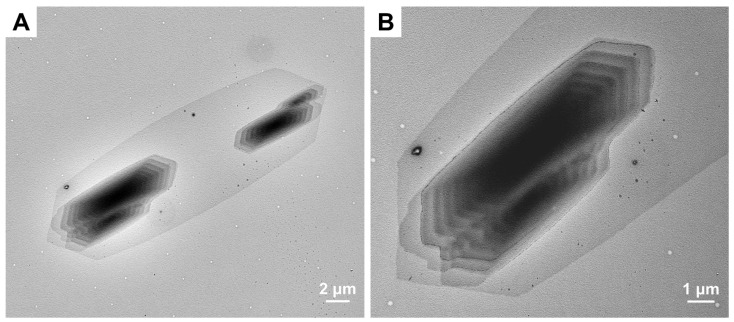
TEM images of PCL_45_-*b*-PSar_28_ self-assemblies prepared in ethanol: (**A**) *T*_s_ = 39.5 °C, *T*_c1_ = 33 °C, *T*_c2_ = 8 °C; (**B**) magnified view of the lamellar crystal.

A preliminary investigation into the self-assembly behavior of PCL_45_-*b*-PSar_28_ in ethanol with the PCL block at a concentration of 0.2 mg/mL was first conducted, focusing on the effect of *T*_c_. Transmission electron microscopy (TEM) characterization reveals that irregular coral-like aggregates are primarily formed by PCL_45_-*b*-PSar_28_, with the termini comprising agglomerated platelet micelles at the *T*_c_s of 25 °C (Figure S3A) and 29 °C (Figure S3B,E). When *T*_c_ increases to 33 °C, multilamellar single crystals containing abundant screw dislocations are formed (Figure S3C,F). Moreover, a large number of spherical micelles are also observed at the higher *T*_c_s of 29 °C and 33 °C, resulting from the competition between micellization and crystallization. Although higher *T*_c_s favor regularly shaped single crystals, slower crystallization kinetics at these elevated *T*_c_s allow concurrent micellization. The micellization, driven by solvophilic/solvophobic interactions, forms amorphous micelles prior to crystallization. Conversely, at lower *T*_c_s, crystallization outpaces micellization, suppressing spherical micelle formation. However, rapid crystallization under these conditions tends to yield irregular crystals, and excessive undercooling may induce micrometer-scale polymer aggregation. In order to achieve uniform self-assemblies of PCL-*b*-PSar, we developed a two-step crystallization strategy (i.e., initial crystallization at a higher *T*_c1_ followed by secondary crystallization at a lower *T*_c2_), as illustrated in Scheme 2. Figure 4 shows the TEM images of PCL_45_-*b*-PSar_28_ self-assemblies prepared in ethanol with *T*_s_ = 39.5 °C, *T*_c1_ = 33 °C, and *T*_c2_ = 8 °C. It is observed that PCL_45_-*b*-PSar_28_ self-assembles into large elongated truncated lozenge-shaped lamellar crystals with screw dislocations, while the majority of spherical micelles disappear at the lower *T*_c2_. In contrast, the self-assembly of PCL_45_-*b*-PSar_28_ via the self-seeding method at a single *T*_c_ = 8 °C yields micrometer-scale irregular aggregates comprising densely stacked lamellae with pronounced morphological defects (Figure S3D). TEM results (Figure 4A and Figure S3C,D) demonstrate that the self-seeding method coupled with the two-step crystallization strategy enables the formation of crystalline structures with superior morphological regularity in PCL_45_-*b*-PSar_28_ compared with the single-step crystallization approach.

**Figure 5:**
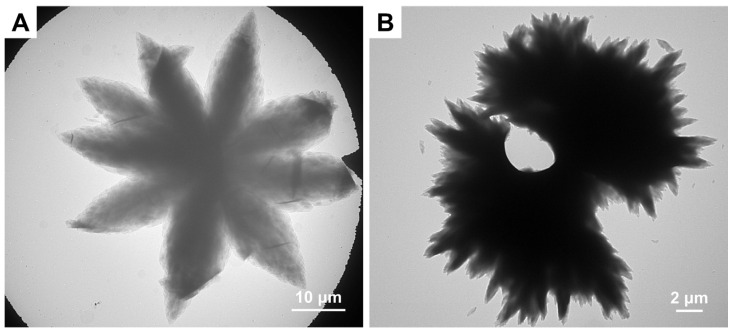
TEM images of self-assemblies of (**A**) PCL_45_-*b*-PSar_43_ and (**B**) PCL_45_-*b*-PSar_81_ prepared in ethanol with *T*_s_ = 39.5 °C, *T*_c1_ = 33 °C, and *T*_c2_ = 8 °C.

Using the self-seeding and two-step crystallization method, the effect of BCP composition of PCL-*b*-PSar on the self-assembled morphology was investigated. Figure 5 displays the TEM images of PCL_45_-*b*-PSar_43_ and PCL_45_-*b*-PSar_81_ self-assemblies (magnified views in Figure S4) prepared under identical crystallization conditions (in ethanol, *T*_s_ = 39.5 °C, *T*_c1_ = 33 °C, *T*_c2_ = 8 °C). PCL_45_-*b*-PSar_43_ forms flower-like aggregates with petal-like lamellae arising from densely packed crystalline sheets (Figure 5A and Figure S4A), while PCL_45_-*b*-PSar_81_ self-assembles into bundled immature spherulites with the characteristic of “fish-eye” (Figure 5B and Figure S4B). With the increase in PSar block length, the self-assembled morphology of the PCL_45_ series BCPs undergoes a transition from two-dimensional (2D) lamellar crystals to three-dimensional (3D) spherulites. Both flower-like aggregates and spherulites represent hierarchical structures evolved from crystalline lamellae, as reported in other semicrystalline BCPs [14,35]. Kang et al. demonstrated that the diblock copolypeptoid poly(*N*-methylglycine)-*b*-poly(*N*-octylglycine) forms hierarchical microflowers via CDSA in methanol [35]. They proposed that such morphology arises from the crystallization-driven fusion and reorganization of initially formed amorphous spherical micelles as primary building blocks, ultimately constructing a flower-like structure composed of radially arranged nanoribbon subunits. Song et al. prepared spherulites of PFS_50_-*b*-P(EG-E)MA_48_ (P(EG-E)MA, poly(tetraethylene glycol monododecyl ether methacrylate)) using a seeded growth approach [14]. The crystalline PFS block tends to form lamellae in the selective solvent decane, whereas the soluble amorphous corona-forming P(EG-E)MA block constrains crystallization directions through steric hindrance, forcing branching growth along specific pathways.

**Figure 6:**
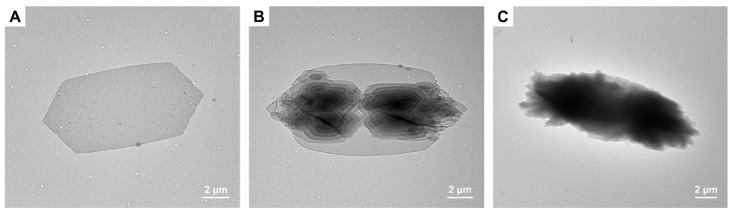
TEM images of self-assemblies of PCL_67_ series BCPs prepared in ethanol with *T*_c1_ = 33 °C and *T*_c2_ = 8 °C: (**A**) PCL_67_-*b*-PSar_34_, *T*_s_ = 43.5 °C; (**B**) PCL_67_-*b*-PSar_69_, *T*_s_ = 42.0 °C; (**C**) PCL_67_-*b*-PSar_99_, *T*_s_ = 41.0 °C.

For the PCL_67_ series, although all three BCPs form 2D morphologies, significant differences in lamellar textural evolution are observed with increasing *DP* of PSar, as shown by the TEM images in Figure 6. PCL_67_-*b*-PSar_34_ self-assembles into monolayer lamellar crystals with elongated truncated lozenge shapes (Figure 6A). In contrast, PCL_67_-*b*-PSar_69_ forms lamellae containing high-density screw dislocations (Figure 6B), while PCL_67_-*b*-PSar_99_ exhibits surface roughening caused by excessive polymer aggregation (Figure 6C). Whereas the PCL_45_ series transitions to 3D spherulites with increasing *DP* of PSar, the PCL_67_ series retains 2D structures at similar weight ratios of PCL/PSar. Our findings reveal that the composition of PCL-*b*-PSar BCPs exerts an important influence on the self-assembled morphology, including the surface feature of the lamellae and the hierarchical structures of the assemblies. BCPs with longer PSar blocks tend to form multilayer lamellae or hierarchical structures. This behavior is probably attributed to the limited solubility of PSar in ethanol, which will be discussed in the subsequent section.

### 2.3. Effect of Solvent Quality on CDSA of PCL-b-PSar

**Figure 7:**
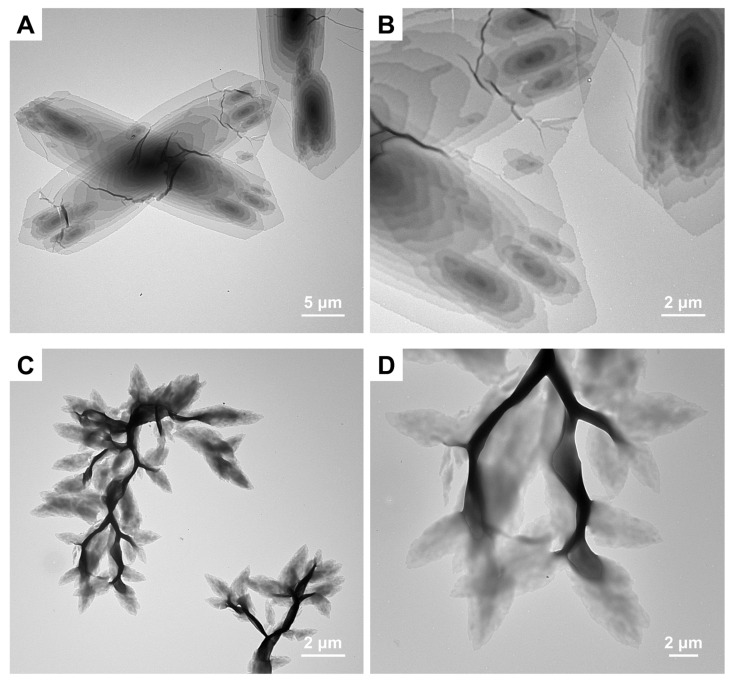
TEM images of PCL_45_-*b*-PSar_28_ self-assemblies prepared in different alcohol solvents with *T*_c1_ = 33 °C and *T*_c2_ = 8 °C: (**A**,**B**) *n*-butanol, *T*_s_ = 39.5 °C; (**C**,**D**) *n*-hexanol, *T*_s_ = 43.5 °C.

The solvent quality can greatly affect the crystallization and micellization rates and thus the self-assembled morphology of semicrystalline BCPs. Herein, CDSA of PCL-*b*-PSar was conducted in different alcohol solvents, including ethanol, *n*-butanol, and *n*-hexanol, since they are frequently used for solution crystallization of PCL homopolymers and PCL-based BCPs [17,26,30,48,72]. The morphologies of PCL_45_-*b*-PSar_28_ prepared in *n*-butanol and *n*-hexanol are shown in Figure 7. While PCL_45_-*b*-PSar_28_ forms multilayer lamellar crystals in *n*-butanol, their crystal surfaces demonstrate more intricate screw dislocations (Figure 7A,B) than those formed in ethanol (Figure 4). In contrast, *n*-hexanol induces the emergence of micrometer-scale dendritic aggregates, a morphology distinct from lamellae. The large aggregates display clear “branch-leaf” architectures, where the “leaves” extending from the “branches” comprise densely packed polymer assemblies (Figure 7C,D). To elucidate the effect of solvent, we determined the Hansen solubility parameters (HSPs), including the dispersion (*δ*_d_), polar (*δ*_p_), and hydrogen bonding (*δ*_h_) components and the total solubility parameter (*δ*_t_), for three alcohol solvents (ethanol, *n*-butanol, and *n*-hexanol) and two homopolymers (PCL and PSar). The solubility parameter distances (*R*_a_) between solvents and polymers were also calculated. These data are presented in Table 2. The solvent HSPs were sourced from Barton’s handbook [73], and the HSPs for PCL were cross-validated using multiple methods against experimental values [74]. The HSPs for PSar were calculated via the Hoftyzer–Van Krevelen group contribution method using Equations (S1)–(S5) [75]. The values of different group contributions originally compiled by Beerbower and presented by Meusburger [73,76] are given in Table S1. The density of PSar was measured as 1.23 g/cm^3^ via Archimedes’ principle (Figure S5).

One can see from Table 2 that the solubility parameter distances between PSar and the three alcohol solvents exceed those for PCL. However, dissolution experiments demonstrate that PSar exhibits superior solubility in all three alcohols relative to PCL (Figures S6−S8). At a polymer concentration of 1 mg/mL and room temperature, PCL_45_ is insoluble in ethanol, *n*-butanol, and *n*-hexanol (Figure S6). In contrast, PSar_51_ is soluble in the three alcohols (Figure S7), while PSar_187_ only dissolves in ethanol (Figure S8). These results indicate that the three alcohols act as poor solvents for PCL, while PSar exhibits moderate solubility in ethanol but limited solubility in longer-chain alcohols. Therefore, during CDSA of PCL-*b*-PSar BCPs in these alcohols, PCL (the insoluble block) constitutes the micellar core, whereas PSar (the soluble block) forms the corona. The relative energy difference (RED = *R*_a_/*R*_0_) predicts solubility better than *R*_a_ alone, where the solubility sphere radius *R*_0_ is polymer-dependent [73]. Thus, the apparent discrepancy between the solubility parameter distances and the experimental dissolution behavior for PSar and PCL may be attributed to PSar’s larger *R*_0_ value. As shown in Table 2, increasing alcohol alkyl chain length markedly reduces *R*_a_ for PCL (43% decrease from ethanol to *n*-hexanol) but gradually increases *R*_a_ for PSar (~8% rise). Since a smaller *R*_a_ value generally indicates better solubility for a given polymer [77], longer hydrocarbon tails in the tested alcohols enhance PCL solubility but reduce PSar solubility. Although *n*-hexanol exhibits closer HSPs to PCL and facilitates the formation of well-defined single crystals in PCL-containing BCPs [30], PSar demonstrates poor solubility in *n*-hexanol due to the significant HSP mismatch (*R*_a,PSar_ = 17.8 MPa^1/2^). Consequently, the aggregation of PCL-*b*-PSar BCPs in *n*-hexanol accelerates PCL crystallization, leading to the formation of an aggregated dendritic structure.

**Scheme 3:**
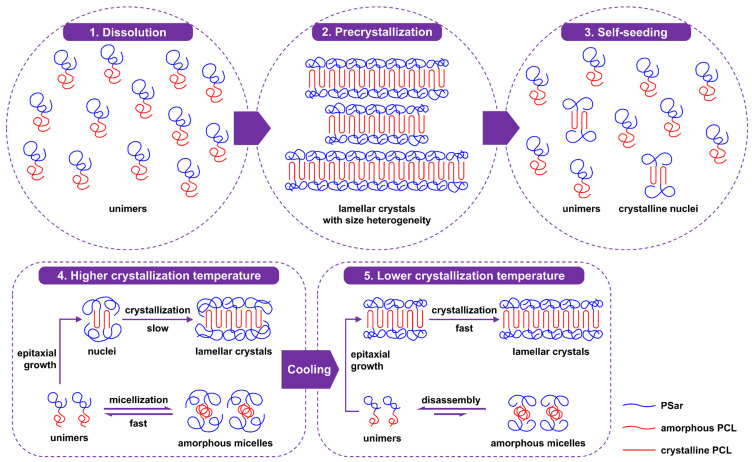
Possible CDSA mechanism of PCL-*b*-PSar BCPs in alcohols via the self-seeding method coupled with the two-step crystallization strategy.

The above results demonstrate the feasibility of the two-step crystallization strategy for preparing regularly shaped crystalline self-assemblies of PCL-*b*-PSar BCPs in alcohol solvents via CDSA, particularly for the BCPs with short PSar blocks in ethanol and *n*-butanol. The possible CDSA mechanism of PCL-*b*-PSar in alcohols can be explained in terms of Scheme 3. Upon cooling the BCP solution from *T*_s_ to the higher *T*_c1_, the soluble PSar block adopts an expanded random-coil conformation. Steric hindrance from the overcrowded amorphous PSar chains tethered on the PCL lamellar surfaces partially shields the lateral crystal surfaces, suppressing epitaxial crystallization at growth fronts. Furthermore, even at the higher *T*_c1_, the solubility of PSar still remains limited, leading to fast micellization that competes with crystallization. As a result, a large number of amorphous micelles coexist with a small quantity of lamellar crystals. Further cooling to the lower *T*_c2_ causes PSar coils to contract, exposing the lateral crystal growth fronts. Moreover, PCL crystallizes faster at this lower temperature. Both these two factors favor rapid lamellar crystal growth. Since crystal growth proceeds via epitaxial attachment of unimers in the solution to the lateral surfaces of the crystals, the consumption of unimers shifts the equilibrium away from amorphous micelles and towards unimers. As crystallization progresses at *T*_c2_, the amorphous micelles disassemble and gradually diminish, leaving behind the regular lamellae. It should be pointed out that the necessity of the two-step crystallization strategy for CDSA of PCL-*b*-PSar BCPs in alcohol solvents, which is different from the single-step crystallization reported for most semicrystalline BCPs in the literature [17,25,30,39,40,48], lies in the limited solubility of PSar. For semicrystalline BCPs with a highly soluble amorphous block, the critical micellization concentration is markedly higher, and the vast majority of BCPs exist as unimers rather than micelles in dilute solution prior to crystallization. Therefore, this two-step crystallization strategy may also be applicable to other semicrystalline BCP/solvent systems where the corona-forming block exhibits limited solubility.

## 3. Materials and Methods

### 3.1. Materials

*ε*-Caprolactone (99%, J&K chemicals) was dried over CaH_2_ (97.0%, Macklin) with stirring overnight at room temperature, followed by purification via vacuum distillation. Diphenyl phosphate (99%, J&K chemicals) was dried under vacuum at 60 °C for 24 h prior to use. Benzyl alcohol (99%, SuperDry solvent, J&K chemicals), toluene (SuperDry solvent, Yonghua chemical), *N*-hydroxyphthalimide (98%, J&K chemicals), triphenylphosphine (99%, J&K chemicals), diisopropyl azodicarboxylate (98%, J&K chemicals), and hydrazine hydrate (85%, AR grade, Sinopharm) were used as received. *N*-Boc-sarcosine (98%, Energy chemical), triphosgene (98%, Energy chemical), 1,2-propylene oxide (99.5%, Sinopharm), acetonitrile (AR grade, Sinopharm), dichloromethane (DCM, 99.9%, SuperDry solvent, J&K chemicals), and glacial acetic acid (99.8%, Sinopharm) were used without further purification. Ethanol (GR grade, Sinopharm), *n*-butanol (GR grade, Sinopharm), *n*-hexanol (98%, J&K chemicals), and all other solvents required no purification.

### 3.2. Characterization Methods

^1^H and ^13^C NMR spectra were recorded on a Bruker AVANCE NEO 400 spectrometer (400 MHz; Bruker Switzerland AG, Fällanden, Switzerland) at room temperature using CDCl_3_ as the solvent and tetramethylsilane as the internal standard. Polydispersity indexes (*Đ*) were determined by a GPC system composed of Waters 1515 isocratic HPLC pump, Waters 2414 refractive index detector, Waters 2707 autosampler, and Styragel series columns (HR3, HR4, and HR5, molecular weights between 500 and 4,000,000) (Waters Corporation, Milford, MA, USA). *N*,*N*-Dimethylformamide containing 0.05 mol/L of LiBr was employed as eluent with a flow rate of 1 mL/min at 60 °C. Commercial poly(methyl methacrylate)s were used as the calibration standards. Turbidity measurements were performed using a UV-2600 UV-vis spectrophotometer (Shimadzu Corporation, Kyoto, Japan). UV-vis absorbance of the samples was recorded at a wavelength of 600 nm. The temperature corresponding to the minimum UV-vis absorbance was determined as the self-seeding temperature (*T*_s_). TEM observations were conducted on a JEOL JEM-1230 transmission electron microscope (JEOL Ltd., Tokyo, Japan) operated at an accelerating voltage of 80 kV. The dispersed samples were dropped onto the carbon-coated copper grids and dried in the air at room temperature.

### 3.3. Synthesis of PCL Macroinitiators

All polymerization reactions were performed using the Schlenk technique, with pre-dried reaction tubes purged with nitrogen. A typical synthetic procedure is described as follows. *ε*-CL (7.1 g, 62 mmol), diphenyl phosphate (0.30 g, 1.2 mmol), and benzyl alcohol (0.14 g, 1.3 mmol) were dissolved in 60 mL of toluene and stirred at 25 °C for 7 h. The polymer solution was precipitated in cold methanol, and the resulting product was dried under vacuum to obtain PCL (6.0 g, yield = 85%). Under a nitrogen atmosphere, PCL (5.2 g, 1.0 mmol) was dissolved in DCM, followed by the addition of *N*-hydroxyphthalimide (0.25 g, 1.5 mmol) and triphenylphosphine (0.40 g, 1.5 mmol). The mixture was cooled in an ice bath, and diisopropyl azodicarboxylate (0.36 mL, 1.8 mmol) was added dropwise slowly. After complete addition, the reaction was allowed to proceed at room temperature for 18 h. The mixture was precipitated in cold methanol, and the resulting precipitate was dried under vacuum to obtain PCL–O–NHP (4.8 g, yield = 93%). Hydrazine hydrate (0.16 g, 2.7 mmol) was added dropwise to a DCM solution of PCL–O–NHP (4.5 g, 0.83 mmol) at room temperature. After stirring for 1 h, the reaction mixture was filtered to remove the white insoluble by-product. The filtrate was precipitated in cold methanol, and the obtained precipitate was dried under vacuum to afford the macroinitiator PCL–O–NH_2_ (2.1 g, yield = 49%). ^1^H NMR of PCL_45_ (400 MHz, CDCl_3_, *δ*, ppm): 1.20–1.47 (90H, –C_2_H_4_C*H*_2_C_2_H_4_–), 1.47–1.82 (180H, –CH_2_C*H*_2_CH_2_C*H*_2_CH_2_–), 2.14–2.46 (90H, –CH_2_COO–), 3.65 (2H, –C*H*_2_OH), 3.86–4.24 (88H, –COOCH_2_–), 5.12 (2H, PhC*H*_2_O–), 7.29–7.40 (5H, *Ph*CH_2_–). ^1^H NMR of PCL_45_–O–NHP (400 MHz, CDCl_3_, *δ*, ppm): 1.17–1.50 (90H, –C_2_H_4_C*H*_2_C_2_H_4_–), 1.50–1.89 (180H, –CH_2_C*H*_2_CH_2_C*H*_2_CH_2_–), 2.10–2.52 (90H, –CH_2_COO–), 3.84–4.29 (90H, –COOCH_2_–, –C*H*_2_–O–NHP), 5.12 (2H, PhC*H*_2_–), 7.29–7.43 (5H, *Ph*CH_2_–), 7.72–7.92 (4H, *N*-substituted phthalimide protons). ^1^H NMR of PCL_45_–O–NH_2_ (400 MHz, CDCl_3_, *δ*, ppm): 1.17–1.49 (90H, –C_2_H_4_C*H*_2_C_2_H_4_–), 1.49–1.85 (180H, –CH_2_C*H*_2_CH_2_C*H*_2_CH_2_–), 2.10–2.50 (90H, –CH_2_COO–), 3.83–4.27 (90H, –COOCH_2_–, –C*H*_2_ONH_2_), 5.12 (2H, PhC*H*_2_–), 7.29–7.40 (5H, *Ph*CH_2_–).

### 3.4. Synthesis of Sar-NCA Monomer

A three-necked flask was placed in an ice bath and successively charged with *N*-Boc sarcosine (10.7 g, 55.5 mmol), acetonitrile (200 mL), and propylene oxide (37 mL, 0.53 mmol). *N*-Boc sarcosine dissolved rapidly under magnetic stirring. Triphosgene (8.2 g, 27 mmol) was added in one portion under a nitrogen atmosphere, and the reaction flask was kept half-open to facilitate the in situ deprotection of Boc. The reaction was stirred at 4 °C in the ice bath for 1.5 h, followed by slow addition of 100 mL cold water to quench excess triphosgene for safety reasons. The mixture was extracted with ethyl acetate (200 mL × 2). The combined organic phase was washed with brine and dried over anhydrous Na_2_SO_4_. After rotary evaporation of the solvent, the crude product was purified by crystallization in cyclohexane/tetrahydrofuran below 10 °C, affording the pure product as a white granular crystal (4.0 g, yield = 63%). ^1^H NMR of Sar-NCA (400 MHz, CDCl_3_, *δ*, ppm): 3.06 (3H, –NCH_3_–), 4.13 (2H, –CH_2_–).

### 3.5. Synthesis of PCL-b-PSar Block Copolymers and PSar Homopolymers

ROP of Sar-NCA was performed using the Schlenk technique. A typical synthesis procedure of PCL-*b*-PSar BCPs is described as follows, while synthesizing PSar homopolymers only requires substituting the PCL–O–NH_2_ macroinitiator with a primary amine initiator. Sar-NCA (0.23 g, 2.0 mmol), DCM (1 mL), and acetic acid (DCM solution, 0.12 M, 3 mL, 0.36 mmol) were sequentially added to a reaction tube. Under magnetic stirring, PCL–O–NH_2_ (DCM solution, 0.011 M, 6 mL, 0.067 mmol) was added to the reaction tube. The polymerization proceeded at room temperature for 30 min with the release of CO_2_. The polymer solution was precipitated in diethyl ether. After centrifugation, the precipitate was dried under vacuum to obtain PCL-*b*-PSar (0.50 g, yield = 99%). ^1^H NMR of PCL_45_-*b*-PSar_28_ (400 MHz, CDCl_3_, *δ*, ppm): 1.20–1.51 (90H, –C_2_H_4_C*H*_2_C_2_H_4_–), 1.51–1.86 (180H, –CH_2_C*H*_2_CH_2_C*H*_2_CH_2_–), 2.12–2.43 (90H, –CH_2_COO–), 2.75–3.25 (84H, –NCH_3_–), 3.75–4.47 (90H, –COOCH_2_–; 56H, –C*H*_2_CONCH_3_–), 5.12 (2H, PhC*H*_2_–), 7.30–7.41 (5H, *Ph*CH_2_–).

### 3.6. Preparation Procedure of PCL-b-PSar Crystalline Micelles

PCL-*b*-PSar crystalline micelles were prepared via the self-seeding method. A typical preparation procedure is described as follows. PCL-*b*-PSar and solvent (ethanol, *n*-butanol, or *n*-hexanol) were added to a test tube at room temperature, with the mass concentration of the PCL block controlled at 0.2 mg/mL. The tube was first placed in a 70 °C oil bath for 2 h to ensure complete dissolution of the BCP. The solution was rapidly quenched to room temperature and allowed to stand overnight to induce crystallization. Subsequently, the tube was maintained at the self-seeding temperature (*T*_s_) for a predetermined time to dissolve most of the polymer crystals, leaving behind a small number of crystalline nuclei for subsequent crystal growth. Finally, the tube was quenched to the first-step isothermal crystallization temperature (*T*_c1_) and maintained for 2 days, followed by quenching to the second-step isothermal crystallization temperature (*T*_c2_) for 3 h of further crystal growth. For single-temperature crystallization experiments, the solution was quenched to the designated isothermal crystallization temperature (*T*_c_) from *T*_s_ and held for 2 days.

### 3.7. Density Determination of PSar Homopolymer

PSar homopolymer was processed into film strips by the solution-casting method, followed by density determination via Archimedes’ principle. PSar_529_ was initially dissolved in deionized water to prepare a 100 mg/mL solution. After ultrasonic defoaming, the solution was cast into a polytetrafluoroethylene mold and dried at ambient temperature to obtain free-standing PSar film strips. Two strips were immersed separately in identical mixed solvent media of isoamyl acetate and 1,2,4-trichlorobenzene. Following ultrasonic treatment to remove bubbles adhered to the surface of the strips, the solvent ratios were adjusted until the strips achieved buoyancy equilibrium, indicating that the solvent density matched the polymer density. Through measurements of the solvent volume and mass, two independent density determinations of 1.223 g/cm^3^ and 1.235 g/cm^3^ were obtained. The arithmetic mean value of 1.23 g/cm^3^ is determined as the definitive density of PSar homopolymer.

## 4. Conclusions

In summary, this study successfully achieved the controlled synthesis of PCL-*b*-PSar BCPs with independently tunable block lengths (*DP* of PCL: 45/67; *DP* of PSar: 28–99) and low polydispersity indexes (*Đ* ≤ 1.1), enabling systematic investigations into their CDSA behavior in alcohol solvents. A key innovation was the development of the self-seeding method by incorporating the two-step crystallization strategy (*T*_c1_ = 33 °C and *T*_c2_ = 8 °C). This modified method overcame limitations of the conventional single-step approach and enhanced morphological regularity, achieving conversion of micelles into crystals and yielding uniform self-assembled structures. The composition of PCL-*b*-PSar affected both the hierarchical assembly structures and the lamellar surface morphologies. Increasing *DP* of the PSar block drove a morphological transition from 2D lamellae to 3D spherulites for the PCL_45_ series BCPs and induced aggregation on crystal surfaces for the PCL_67_ series. Solvent quality notably dictated the self-assembly pathways. PCL_45_-*b*-PSar_28_ formed 2D lamellae in ethanol and *n*-butanol but generated micrometer-scale dendritic aggregates in *n*-hexanol. The HSP analysis and the dissolution experiments revealed that PSar exhibited limited solubility in alcohols; thus, a large number of amorphous micelles coexisted with lamellar crystals at the higher *T*_c1_. At the lower *T*_c2_, fast growth of the lamellar crystallization via attachment of unimers triggered micelle disassembly. The amphiphilic PCL-*b*-PSar BCPs demonstrate excellent biocompatibility and biodegradability. Significantly, the well-defined 2D lamellae formed via CDSA possess distinct functional advantages, such as enhanced drug-loading capacity and potential for directional delivery. These attributes position the BCP as a promising candidate for advanced drug delivery vehicles and theranostic platforms. Building upon the mechanistic insights into micellization-crystallization competition, future studies will extend this modified CDSA approach to other BCP systems while concurrently evaluating the biomedical potential of the obtained 2D lamellae in drug delivery and theranostics.

## Figures and Tables

**Table 1:** Structural characteristics of PCL homopolymers and PCL-*b*-PSar BCPs.

Composition ^a^	*M*_n,NMR_ ^b^ (kg/mol)	*Đ* ^c^	Weight Ratio of PCL/PSar ^d^
PCL_45_	5.2	1.04	-
PCL_67_	7.8	1.05	-
PCL_45_-*b*-PSar_28_	7.2	1.06	72/28
PCL_45_-*b*-PSar_43_	8.3	1.06	63/37
PCL_45_-*b*-PSar_81_	11.0	1.06	47/52
PCL_67_-*b*-PSar_34_	10.2	1.07	76/24
PCL_67_-*b*-PSar_69_	12.7	1.06	61/39
PCL_67_-*b*-PSar_99_	14.8	1.10	52/48

^a^ Determined by ^1^H NMR. ^b^ For PCL homopolymers: $M_{n,NMR}^{PCL}={DP}_{PCL}\times 114.14\;g/mol+M_{initiator}$, where $M_{initiator}=108.14\;g/mol$ (molecular weight of benzyl alcohol); for PCL-*b*-PSar BCPs: $M_{n,NMR}^{BCP}={DP}_{PCL}\times 114.14\;g/mol+{DP}_{PSar}\times 71.08\;g/mol+M_{ends+junction}$, where $M_{ends+junction}=123.16\;g/mol$ (total molecular weight of the end groups and the junction moiety). ^c^ Determined by GPC and calibrated with PMMA standards. ^d^ Calculated by $\left({DP}_{PCL}\times 114.14\right)/\left({DP}_{PSar}\times 71.08\right)$.

**Table 2:** Hansen solubility parameters of solvents and polymers.

Category	Material	*δ*_d_/MPa^1/2^	*δ*_p_/MPa^1/2^	*δ*_h/_MPa^1/2^	*δ*_t_ ^a^/MPa^1/2^	*R*_a,PCL_ ^b^/MPa^1/2^	*R*_a,PSar_ ^b^/MPa^1/2^
Solvents	Ethanol ^c^	15.8	8.8	19.4	26.5	12.5	16.4
	*n*-Butanol ^c^	16.0	5.7	15.8	23.2	8.7	17.1
	*n*-Hexanol ^c^	15.8	4.3	13.5	21.2	7.1	17.8
Polymers	PCL ^d^	17.7	6.2	7.8	20.3	-	-
	PSar ^e^	17.1	21.3	9.1	28.8	-	-

^a^ $\delta_{t}={\left(\delta_{d}^{2}+\delta_{p}^{2}+\delta_{h}^{2}\right)}^{1/2}$. ^b^ $R_{a}={\left[4{\left(\delta_{d,S}-\delta_{d,P}\right)}^{2}+{\left(\delta_{p,S}-\delta_{p,P}\right)}^{2}+{\left(\delta_{h,S}-\delta_{h,P}\right)}^{2}\right]}^{1/2}$. The subscripts S and P represent solvent and polymer, respectively. ^c^ Data from [73] ^d^ Data from [74] ^e^ Calculated according to [75].

## Data Availability

The original contributions presented in this study are included in the article and Supplementary Materials. Further inquiries can be directed to the corresponding authors.

## Supplementary Materials

The following supporting information can be downloaded at: https://www.mdpi.com/article/10.3390/molecules30153108/s1, Figure S1: ^1^H NMR spectrum of Sar-NCA in CDCl_3_; Figure S2: Turbidity of PCL_45_ series block copolymers in ethanol at different temperatures, with the mass concentration of the PCL block controlled at 0.2 mg/mL; Figure S3: TEM images of PCL_45_-*b*-PSar_28_ self-assemblies prepared in ethanol with *T*_s_ = 39.5 °C and different *T*_c_s: (A) *T*_c_ = 25 °C; (B,E) *T*_c_ = 29 °C; (C,F) *T*_c_ = 33 °C; (D) *T*_c_ = 8 °C. Inset enlargements within the large white frames show zoomed-in views of the regions marked by the small white frames in (C) and (E), respectively, highlighting the spherical micelle; Figure S4: High-magnification TEM images of self-assemblies of (A) PCL_45_-*b*-PSar_43_ and (B) PCL_45_-*b*-PSar_81_ prepared in ethanol with *T*_s_ = 39.5 °C, *T*_c1_ = 33 °C, and *T*_c2_ = 8 °C; Figure S5: Density determination of PSar via neutral buoyancy. PSar_529_ specimen suspended in a mixed solvent of isoamyl acetate and 1,2,4-trichlorobenzene, enabling density calculation (*ρ*_PSar_ = 1.23 g/cm^3^) based on Archimedes’ principle; Table S1: Group contributions for Hansen solubility parameters compiled by Beerbower; Equations (S1)−(S5): Calculation of Hansen solubility parameters for PSar; Figure S6−S8: Visual assessment of solubility behavior of PCL_45_, PSar_51_, and PSar_187_ (1 mg/mL). Row order: ethanol, *n*-butanol, *n*-hexanol; Column order: room temperature mixing, 70 °C heating for 1 h, room temperature cooling for 12 h.

## References

[1] Yu P.J., Lin Y.C., Chen W.C. (2025). Review of bioderived and biodegradable polymers/block-copolymers and their biomedical and electronic applications. Polym. J..

[2] Mai Y.Y., Eisenberg A. (2012). Self-assembly of block copolymers. Chem. Soc. Rev..

[3] Holder S.J., Sommerdijk N.A.J.M. (2011). New micellar morphologies from amphiphilic block copolymers: Disks, toroids and bicontinuous micelles. Polym. Chem..

[4] Lu Y.Q., Lin J.P., Wang L.Q., Zhang L.S., Cai C.H. (2020). Self-assembly of copolymer micelles: Higher-level assembly for constructing hierarchical structure. Chem. Rev..

[5] MacFarlane L., Zhao C.Q., Cai J.D., Qiu H.B., Manners I. (2021). Emerging applications for living crystallization-driven self-assembly. Chem. Sci..

[6] Ganda S., Stenzel M.H. (2020). Concepts, fabrication methods and applications of living crystallization-driven self-assembly of block copolymers. Prog. Polym. Sci..

[7] Zhang T.Y., Xu J.T. (2019). One-dimensional growth kinetics for formation of cylindrical crystalline micelles of block copolymers. Polym. Cryst..

[8] Yang C., Li Z.X., Xu J.T. (2022). Single crystals and two-dimensional crystalline assemblies of block copolymers. J. Polym. Sci..

[9] He W.N., Xu J.T. (2012). Crystallization assisted self-assembly of semicrystalline block copolymers. Prog. Polym. Sci..

[10] Massey J., Power K.N., Manners I., Winnik M.A. (1998). Self-assembly of a novel organometallic−inorganic block copolymer in solution and the solid State:  Nonintrusive observation of novel wormlike poly(ferrocenyldimethylsilane)-*b*-poly(dimethylsiloxane) micelles. J. Am. Chem. Soc..

[11] Wang X.S., Guerin G., Wang H., Wang Y.S., Manners I., Winnik M.A. (2007). Cylindrical block copolymer micelles and co-micelles of controlled length and architecture. Science.

[12] Gilroy J.B., Gädt T., Whittell G.R., Chabanne L., Mitchels J.M., Richardson R.M., Winnik M.A., Manners I. (2010). Monodisperse cylindrical micelles by crystallization-driven living self-assembly. Nat. Chem..

[13] Jiang J.J., Nikbin E., Plaha T.S., Howe J.Y., Winnik M.A. (2024). Crystallization-driven self-assembly of polyferrocenyldimethylsilane block copolymers with the corona modified with pendant amino groups. Macromolecules.

[14] Song S.F., Zhou H., Ye S.Y., Tam J., Howe J.Y., Manners I., Winnik M.A. (2021). Spherulite-like micelles. Angew. Chem. Int. Ed..

[15] Xu J.T., Fairclough J.P.A., Mai S.M., Ryan A.J. (2003). The effect of architecture on the morphology and crystallization of oxyethylene/oxybutylene block copolymers from micelles in *n*-hexane. J. Mater. Chem..

[16] Mihut A.M., Drechsler M., Möller M., Ballauff M. (2010). Sphere-to-rod transition of micelles formed by the semicrystalline polybutadiene-*block*-poly(ethylene oxide) block copolymer in a selective solvent. Macromol. Rapid Commun..

[17] Van Horn R.M., Zheng J.X., Sun H.J., Hsiao M.S., Zhang W.B., Dong X.H., Xu J.T., Thomas E.L., Lotz B., Cheng S.Z.D. (2010). Solution crystallization behavior of crystalline−crystalline diblock copolymers of poly(ethylene oxide)-*block*-poly(*ε*-caprolactone). Macromolecules.

[18] Schmelz J., Schedl A.E., Steinlein C., Manners I., Schmalz H. (2012). Length control and block-type architectures in worm-like micelles with polyethylene cores. J. Am. Chem. Soc..

[19] Yin L.G., Lodge T.P., Hillmyer M.A. (2012). A stepwise “micellization–crystallization” route to oblate ellipsoidal, cylindrical, and bilayer micelles with polyethylene cores in water. Macromolecules.

[20] Fan B., Wang R.Y., Wang X.Y., Xu J.T., Du B.Y., Fan Z.Q. (2017). Crystallization-driven co-assembly of micrometric polymer hybrid single crystals and nanometric crystalline micelles. Macromolecules.

[21] Fan B., Liu L., Li J.H., Ke X.X., Xu J.T., Du B.Y., Fan Z.Q. (2016). Crystallization-driven one-dimensional self-assembly of polyethylene-*b*-poly(*tert*-butylacrylate) diblock copolymers in DMF: Effects of crystallization temperature and the corona-forming block. Soft Matter.

[22] Inam M., Jones J.R., Pérez-Madrigal M.M., Arno M.C., Dove A.P., O’Reilly R.K. (2018). Controlling the size of two-dimensional polymer platelets for water-in-water emulsifiers. ACS Cent. Sci..

[23] Inam M., Cambridge G., Pitto-Barry A., Laker Z.P.L., Wilson N.R., Mathers R.T., Dove A.P., O’Reilly R.K. (2017). 1D vs. 2D shape selectivity in the crystallization-driven self-assembly of polylactide block copolymers. Chem. Sci..

[24] Li Z., Zhang Y.F., Wu L.B., Yu W., Wilks T.R., Dove A.P., Ding H.M., O’Reilly R.K., Chen G.S., Jiang M. (2019). Glyco-platelets with controlled morphologies via crystallization-driven self-assembly and their shape-dependent interplay with macrophages. ACS Macro Lett..

[25] Zhang T.Y., Guo X.S., Zhang Z.K., Xu J.T., Fan Z.Q. (2020). Solution-grown composite single crystals of poly(L-lactic acid)-*b*-polystyrene block copolymers and poly(L-lactic acid) homopolymers. Polymer.

[26] Liu L.P., Meng X.C., Li M.L., Chu Z.Y., Tong Z.Z. (2024). Regulation of two-dimensional platelet micelles with tunable core composition distribution via coassembly seeded growth approach. ACS Macro Lett..

[27] Wang J., Lu Y., Chen Y.M. (2019). Fabrication of 2D surface-functional polymer platelets via crystallization-driven self-assembly of poly(*ε*-caprolactone)-contained block copolymers. Polymer.

[28] Su Y.W., Jiang Y.K., Liu L.P., Xie Y.J., Chen S.C., Wang Y.J., O’Reilly R.K., Tong Z.Z. (2022). Hydrogen-bond-regulated platelet micelles by crystallization-driven self-assembly and templated growth for poly(*ε*-caprolactone) block copolymers. Macromolecules.

[29] Xie Y.J., Tong Z.Z., Xia T.L., Worch J.C., Rho J.Y., Dove A.P., O’Reilly R.K. (2024). 2D hierarchical microbarcodes with expanded storage capacity for optical multiplex and information encryption. Adv. Mater..

[30] Zhuo W.Q., Li Y.M., Zhang R.K., Huang R.S., Zhou J., Tong Z.Z., Jiang G.H. (2017). Single crystals of crystalline block copolymers formed in *n*-hexanol and methanol/DMF solutions: A comparative study. J. Appl. Polym. Sci..

[31] Du Z.X., Xu J.T., Fan Z.Q. (2007). Micellar morphologies of poly(*ε*-caprolactone)-*b*-poly(ethylene oxide) block copolymers in water with a crystalline core. Macromolecules.

[32] He W.N., Zhou B., Xu J.T., Du B.Y., Fan Z.Q. (2012). Two growth modes of semicrystalline cylindrical poly(*ε*-caprolactone)-*b*-poly(ethylene oxide) micelles. Macromolecules.

[33] Lee C.U., Smart T.P., Guo L., Epps T.H., Zhang D.H. (2011). Synthesis and characterization of amphiphilic cyclic diblock copolypeptoids from *N*-heterocyclic carbene-mediated zwitterionic polymerization of *N*-substituted *N*-carboxyanhydride. Macromolecules.

[34] Wang Z.W., Lin M., Bonduelle C., Li R.Y., Shi Z.K., Zhu C.H., Lecommandoux S., Li Z.B., Sun J. (2020). Thermoinduced crystallization-driven self-assembly of bioinspired block copolymers in aqueous solution. Biomacromolecules.

[35] Kang L.Y., Chao A., Zhang M., Yu T.Y., Wang J., Wang Q., Yu H.H., Jiang N.S., Zhang D.H. (2021). Modulating the molecular geometry and solution self-assembly of amphiphilic polypeptoid block copolymers by side chain branching pattern. J. Am. Chem. Soc..

[36] Shi B.Y., Shen D., Li W., Wang G.W. (2022). Self-assembly of copolymers containing crystallizable blocks: Strategies and applications. Macromol. Rapid Commun..

[37] Cortes M.D.L.A., de la Campa R., Valenzuela M.L., Díaz C., Carriedo G.A., Presa Soto A. (2019). Cylindrical micelles by the self-assembly of crystalline-*b*-coil polyphosphazene-*b*-P2VP block copolymers. Stabilization of gold nanoparticles. Molecules.

[38] Folgado E., Mayor M., Ladmiral V., Semsarilar M. (2020). Evaluation of self-assembly pathways to control crystallization-driven self-assembly of a semicrystalline P(VDF-*co*-HFP)-*b*-PEG-*b*-P(VDF-*co*-HFP) triblock copolymer. Molecules.

[39] Qian J.S., Li X.Y., Lunn D.J., Gwyther J., Hudson Z.M., Kynaston E., Rupar P.A., Winnik M.A., Manners I. (2014). Uniform, high aspect ratio fiber-like micelles and block co-micelles with a crystalline *π*-conjugated polythiophene core by self-seeding. J. Am. Chem. Soc..

[40] Tao D.L., Feng C., Lu Y.J., Cui Y.N., Yang X., Manners I., Winnik M.A., Huang X.Y. (2018). Self-seeding of block copolymers with a *π*-conjugated oligo(*p*-phenylenevinylene) segment: A versatile route toward monodisperse fiber-like nanostructures. Macromolecules.

[41] Taghizadeh F., Heidari M., Mostafavi S., Mortazavi S.M., Haeri A. (2024). A review of preparation methods and biomedical applications of poly(*ε*-caprolactone)-based novel formulations. J. Mater. Sci..

[42] Ibrahim M., Ramadan E., Elsadek N.E., Emam S.E., Shimizu T., Ando H., Ishima Y., Elgarhy O.H., Sarhan H.A., Hussein A.K. (2022). Polyethylene glycol (PEG): The nature, immunogenicity, and role in the hypersensitivity of PEGylated products. J. Control. Release.

[43] Jiang N., Jiang S.D., Hou Y., Yan S.K., Zhang G.Z., Gan Z.H. (2010). Influence of chemical structure on enzymatic degradation of single crystals of PCL-*b*-PEO amphiphilic block copolymer. Polymer.

[44] Zhu W., Peng B., Wang J., Zhang K., Liu L.X., Chen Y.M. (2014). Bamboo leaf-like micro-nano sheets self-assembled by block copolymers as wafers for cells. Macromol. Biosci..

[45] He W.N., Xu J.T., Du B.Y., Fan Z.Q., Sun F.L. (2012). Effect of pH on the micellar morphology of semicrystalline PCL-*b*-PEO block copolymers in aqueous solution. Macromol. Chem. Phys..

[46] He W.N., Xu J.T., Du B.Y., Fan Z.Q., Wang X.S. (2010). Inorganic-salt-induced morphological transformation of semicrystalline micelles of PCL-*b*-PEO block copolymer in aqueous solution. Macromol. Chem. Phys..

[47] Rizis G., van de Ven T.G.M., Eisenberg A. (2014). “Raft” formation by two-dimensional self-assembly of block copolymer rod micelles in aqueous solution. Angew. Chem. Int. Ed..

[48] Yang C., Li Z.X., Xu J.T. (2024). Hybrid single crystals of poly(*ε*-caprolactone) homopolymers and poly(*ε*-caprolactone)-*b*-poly(ethylene oxide) block copolymers. Macromol. Chem. Phys..

[49] d’Avanzo N., Celia C., Barone A., Carafa M., Di Marzio L., Santos H.A., Fresta M. (2020). Immunogenicity of polyethylene glycol based nanomedicines: Mechanisms, clinical implications and systematic approach. Adv. Ther..

[50] Elsadek N.E., Abu Lila A.S., Ishida T., Pasut G., Zalipsky S. (2020). 5—Immunological responses to PEGylated proteins: Anti-PEG antibodies. Polymer-Protein Conjugates.

[51] Turecek P.L., Siekmann J., Pasut G., Zalipsky S. (2020). 4—PEG–protein conjugates: Nonclinical and clinical toxicity considerations. Polymer-Protein Conjugates.

[52] Tang Y.C., Deber C.M. (2002). Hydrophobicity and helicity of membrane-interactive peptides containing peptoid residues. Biopolymers.

[53] Ostuni E., Chapman R.G., Holmlin R.E., Takayama S., Whitesides G.M. (2001). A survey of structure−property relationships of surfaces that resist the adsorption of protein. Langmuir.

[54] Birke A., Ling J., Barz M. (2018). Polysarcosine-containing copolymers: Synthesis, characterization, self-assembly, and applications. Prog. Polym. Sci..

[55] Zhu H., Chen Y., Yan F.J., Chen J., Tao X.F., Ling J., Yang B., He Q.J., Mao Z.W. (2017). Polysarcosine brush stabilized gold nanorods for in vivo near-infrared photothermal tumor therapy. Acta Biomater..

[56] Hu M.X., Taguchi K., Matsumoto K., Kobatake E., Ito Y., Ueda M. (2023). Polysarcosine-coated liposomes attenuating immune response induction and prolonging blood circulation. J. Colloid. Interf. Sci..

[57] Yao X.K., Sun C.R., Xiong F., Zhang W.N., Yao W.J., Xu Y.H., Fan W.P., Huo F.W. (2024). Polysarcosine as PEG alternative for enhanced camptothecin-induced cancer immunogenic cell death. ACS Appl. Mater. Interfaces.

[58] Hu Y.L., Hou Y.Q., Wang H., Lu H. (2018). Polysarcosine as an alternative to PEG for therapeutic protein conjugation. Bioconjugate Chem..

[59] Cui S.D., Wang X., Li Z.J., Zhang Q.G., Wu W.Z., Liu J.J., Wu H., Chen C., Guo K. (2014). One-pot glovebox-free synthesis, characterization, and self-assembly of novel amphiphilic poly(sarcosine-*b*-caprolactone) diblock copolymers. Macromol. Rapid. Commun..

[60] Cui S.D., Pan X.F., Gebru H., Wang X., Liu J.Q., Liu J.J., Li Z.J., Guo K. (2017). Amphiphilic star-shaped poly(sarcosine)-*block*-poly(*ε*-caprolactone) diblock copolymers: One-pot synthesis, characterization, and solution properties. J. Mater. Chem. B.

[61] Deng Y.W., Zou T., Tao X.F., Semetey V., Trepout S., Marco S., Ling J., Li M.H. (2015). Poly(*ε*-caprolactone)-*block*-polysarcosine by ring-opening polymerization of sarcosine *N*-thiocarboxyanhydride: Synthesis and thermoresponsive self-assembly. Biomacromolecules.

[62] Lv R.K., Qian Z.Z., Zhao X.P., Xiong F., Xu Y.J., Fan W.P., Yao X.K., Huang W. (2023). Self-assembly of polysarcosine amphiphilic polymers-tethered gold nanoparticles for precise photo-controlled synergistic therapy. Nano Res..

[63] Morrell A.H., Warren N.J., Thornton P.D. (2024). The production of polysarcosine-containing nanoparticles by ring-opening polymerization-induced self-assembly. Macromol. Rapid. Commun..

[64] Makiguchi K., Satoh T., Kakuchi T. (2011). Diphenyl phosphate as an efficient cationic organocatalyst for controlled/living ring-opening polymerization of *δ*-valerolactone and *ε*-caprolactone. Macromolecules.

[65] Kricheldorf H.R. (2006). Polypeptides and 100 years of chemistry of *α*-amino acid *N*-carboxyanhydrides. Angew. Chem. Int. Ed..

[66] Tao X.F., Zheng B.T., Bai T.W., Zhu B.K., Ling J. (2017). Hydroxyl group tolerated polymerization of *N*-substituted glycine *N*-thiocarboxyanhydride mediated by aminoalcohols: A simple way to *α*-hydroxyl-*ω*-aminotelechelic polypeptoids. Macromolecules.

[67] Schlick T.L., Ding Z.B., Kovacs E.W., Francis M.B. (2005). Dual-surface modification of the tobacco mosaic virus. J. Am. Chem. Soc..

[68] Novoa-Carballal R., Müller A.H.E. (2012). Synthesis of polysaccharide-*b*-PEG block copolymers by oxime click. Chem. Commun..

[69] Tian Z.Y., Zhang Z.C., Wang S., Lu H. (2021). A moisture-tolerant route to unprotected *α*/*β*-amino acid *N*-carboxyanhydrides and facile synthesis of hyperbranched polypeptides. Nat. Commun..

[70] Wang S., Lu M.Y., Wan S.K., Lyu C.Y., Tian Z.Y., Liu K., Lu H. (2024). Precision synthesis of polysarcosine via controlled ring-opening polymerization of *N*-carboxyanhydride: Fast kinetics, ultrahigh molecular weight, and mechanistic insights. J. Am. Chem. Soc..

[71] Blundell D.J., Keller A., Kovacs A.J. (1966). A new self-nucleation phenomenon and its application to growing of polymer crystals from solution. J. Polym. Sci. Part B Polym. Lett..

[72] Núñez E., Gedde U.W. (2005). Single crystal morphology of star-branched polyesters with crystallisable poly(*ε*-caprolactone) arms. Polymer.

[73] Barton A.F.M. (1991). CRC Handbook of Solubility Parameters and Other Cohesion Parameters.

[74] Bordes C., Fréville V., Ruffin E., Marote P., Gauvrit J.Y., Briançon S., Lantéri P. (2010). Determination of poly(*ε*-caprolactone) solubility parameters: Application to solvent substitution in a microencapsulation process. Int. J. Pharm..

[75] van Krevelen D.W., te Nijenhuis K. (2009). Properties of Polymers: Their Correlation with Chemical Structure; Their Numerical Estimation and Prediction from Additive Group Contributions.

[76] Meusburger K.E., Cross B., Scher H.B. (1988). Determination of cohesive energy density parameters for developing pesticide formulations. Pesticide Formulations: Innovations and Developments.

[77] Hansen C.M. (2007). Hansen Solubility Parameters: A User’s Handbook.

