# Supplementary material for: Controlled Synthesis and Crystallization-Driven Self-Assembly of Poly(ε-caprolactone)-b-polysarcosine Block Copolymers
Source: Molecules. 2025 Jul 24;30(15):3108. doi: 10.3390/molecules30153108 (PMC12348651; doi:10.3390/molecules30153108)
Supplement: Supplementary file 1 [file molecules-30-03108-s001.zip › molecules-3746664-supplementary for XML.pdf]

Supplementary Materials

Controlled Synthesis and Crystallization-Driven Self-Assembly of Poly(*ε*-caprolactone)-*b*-Polysarcosine Block Copolymers

Zi-Xian Li[1], Chen Yang[1], Lei Guo[1], Jun Ling[2], Jun-Ting Xu[1,*]

1 State Key Laboratory of Biobased Transportation Fuel Technology, Department of Polymer Science and Engineering, Zhejiang University, Hangzhou 310058, China

2 MOE Key Laboratory of Macromolecular Synthesis and Functionalization, Department of Polymer Science and Engineering, Zhejiang University, Hangzhou 310058, China

Contents:

Part 1: Characterization of Sar-NCA monomer: S2

Part 2: Determination of self-seeding temperatures (T_s) for PCL_{45} series BCPs: S3

Part 3: TEM images of self-assemblies of PCL_{45} series BCPs: S4

Part 4: Density determination of PSar: S5

Part 5: Determination of Hansen solubility parameters for PSar: S6

Part 6: Visual assessment of solubility behavior of PCL and PSar: S7

Part 1: Characterization of Sar-NCA monomer

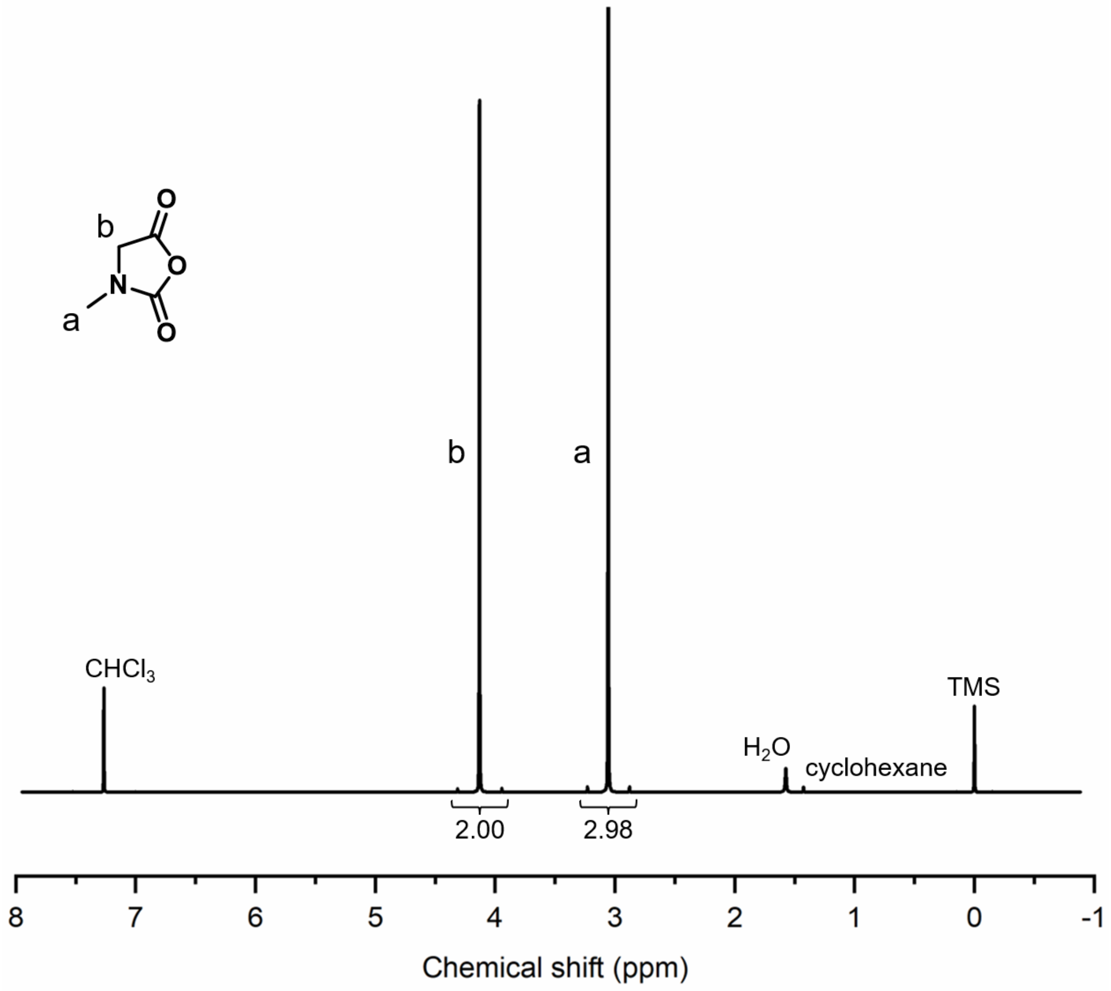

Figure S1. ^{1}H NMR spectrum of Sar-NCA in $CDCl_3$.

Part 2: Determination of self-seeding temperatures (T_s) for PCL_{45} series BCPs

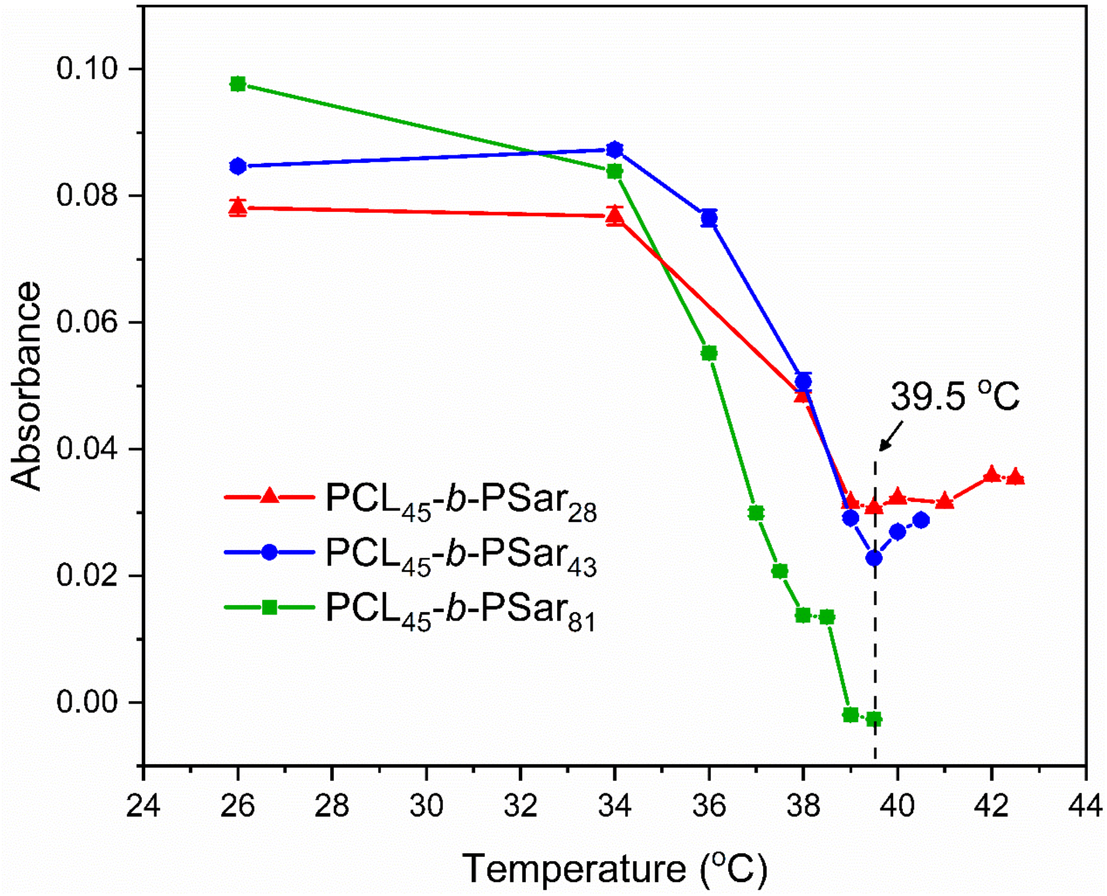

Figure S2. Turbidity of PCL_{45} series block copolymers in ethanol at different temperatures, with the mass concentration of the PCL block controlled at 0.2 mg/mL.

Part 3: TEM images of self-assemblies of PCL_{45} series BCPs

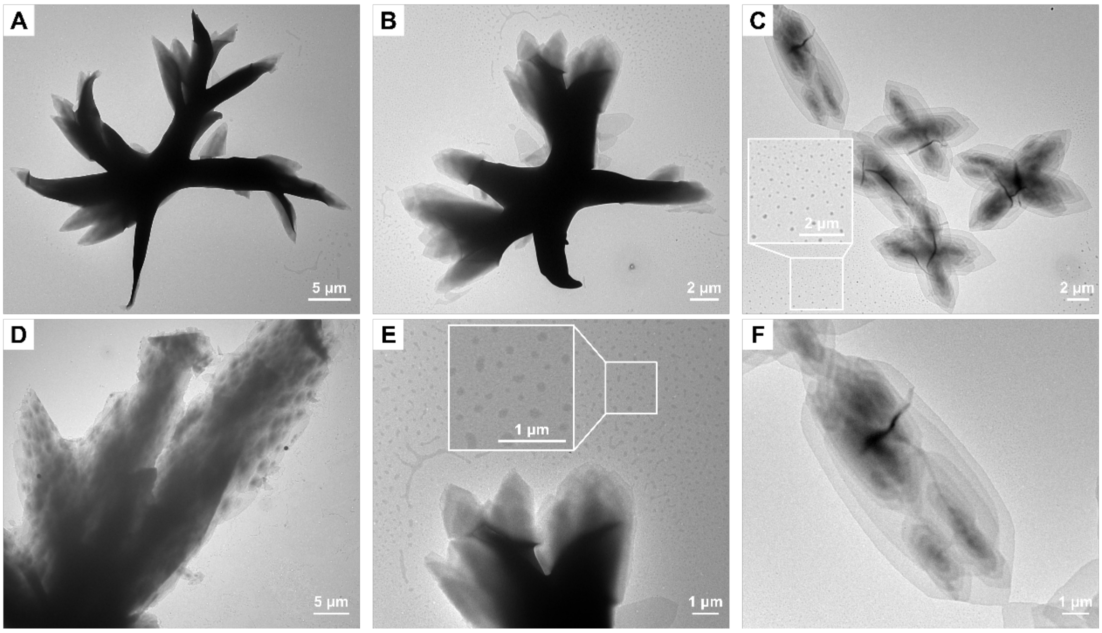

Figure S3. TEM images of PCL_{45}-*b*-$PSar_{28}$ self-assemblies prepared in ethanol with T_s = 39.5 °C and different T_cs: (A) T_c = 25 °C; (B,E) T_c = 29 °C; (C,F) T_c = 33 °C; (D) T_c = 8 °C. Inset enlargements within the large white frames show zoomed-in views of the regions marked by the small white frames in (C) and (E), respectively, highlighting the spherical micelles.

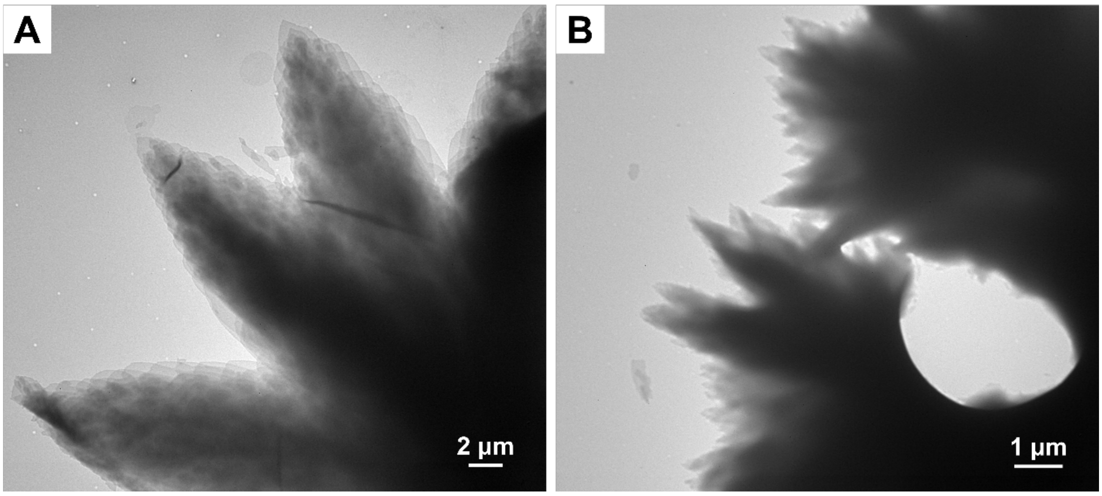

Figure S4. High-magnification TEM images of self-assemblies of (A) PCL_{45}-*b*-$PSar_{43}$ and (B) PCL_{45}-*b*-$PSar_{81}$ prepared in ethanol with T_s = 39.5 °C, T_{c1} = 33 °C, and T_{c2} = 8 °C.

Part 4: Density determination of PSar

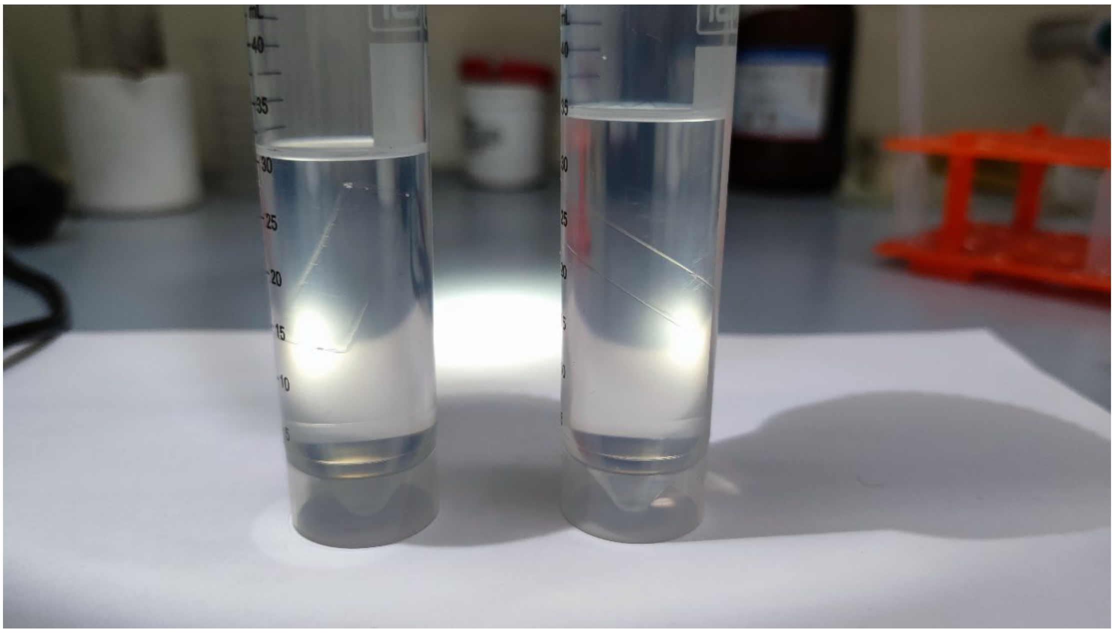

Figure S5. Density determination of PSar via neutral buoyancy. $PSar_{529}$ specimen suspended in a mixed solvent of isoamyl acetate and 1,2,4-trichlorobenzene, enabling density calculation (ρ_{PSar} = 1.23 g/cm^3) based on Archimedes' principle.

Part 5: Determination of Hansen solubility parameters for PSar

Table S1. Group contributions for Hansen solubility parameters compiled by Beerbower

Group	F_{di} / $(MJ/m^3)^{1/2}\cdot mol^{-1}$	F_{pi} / $(MJ/m^3)^{1/2}\cdot mol^{-1}$	E_{hi} / $J\cdot mol^{-1}$
$-CH_3$	419	0	0
$-CH_2$	270	0	0
−CON<	301	1229	4772

The molar volume V of the PSar repeating unit is:

$$V_{\mathrm{Sar}} = \frac{M_{\mathrm{Sar}}}{\rho_{\mathrm{PSar}}} = \frac{71.08\ \mathrm{g/mol}}{1.23\ \mathrm{g/cm^3}} = 57.8\ \mathrm{cm^3/mol} \qquad \text{(S1)}$$

$$\delta_{\mathrm{d,PSar}} = \frac{\sum F_{\mathrm{di}}}{V_{\mathrm{Sar}}} = \frac{419 + 270 + 301}{57.8}\ \mathrm{MPa^{1/2}} = 17.1\ \mathrm{MPa^{1/2}} \qquad \text{(S2)}$$

$$\delta_{\mathrm{p,PSar}} = \frac{\sqrt{\sum F_{\mathrm{pi}}^2}}{V_{\mathrm{Sar}}} = \frac{\sqrt{1229^2}}{57.8}\ \mathrm{MPa^{1/2}} = 21.3\ \mathrm{MPa^{1/2}} \qquad \text{(S3)}$$

$$\delta_{\mathrm{h,PSar}} = \sqrt{\frac{\sum E_{\mathrm{hi}}}{V_{\mathrm{Sar}}}} = \sqrt{\frac{4772}{57.8}}\ \mathrm{MPa^{1/2}} = 9.1\ \mathrm{MPa^{1/2}} \qquad \text{(S4)}$$

$$\delta_{\mathrm{t,PSar}} = \sqrt{\delta_{\mathrm{d}}^2 + \delta_{\mathrm{p}}^2 + \delta_{\mathrm{h}}^2} = \sqrt{17.1^2 + 21.3^2 + 9.1^2}\ \mathrm{MPa^{1/2}} = 28.8\ \mathrm{MPa^{1/2}} \qquad \text{(S5)}$$

Part 6: Visual assessment of solubility behavior of PCL and PSar

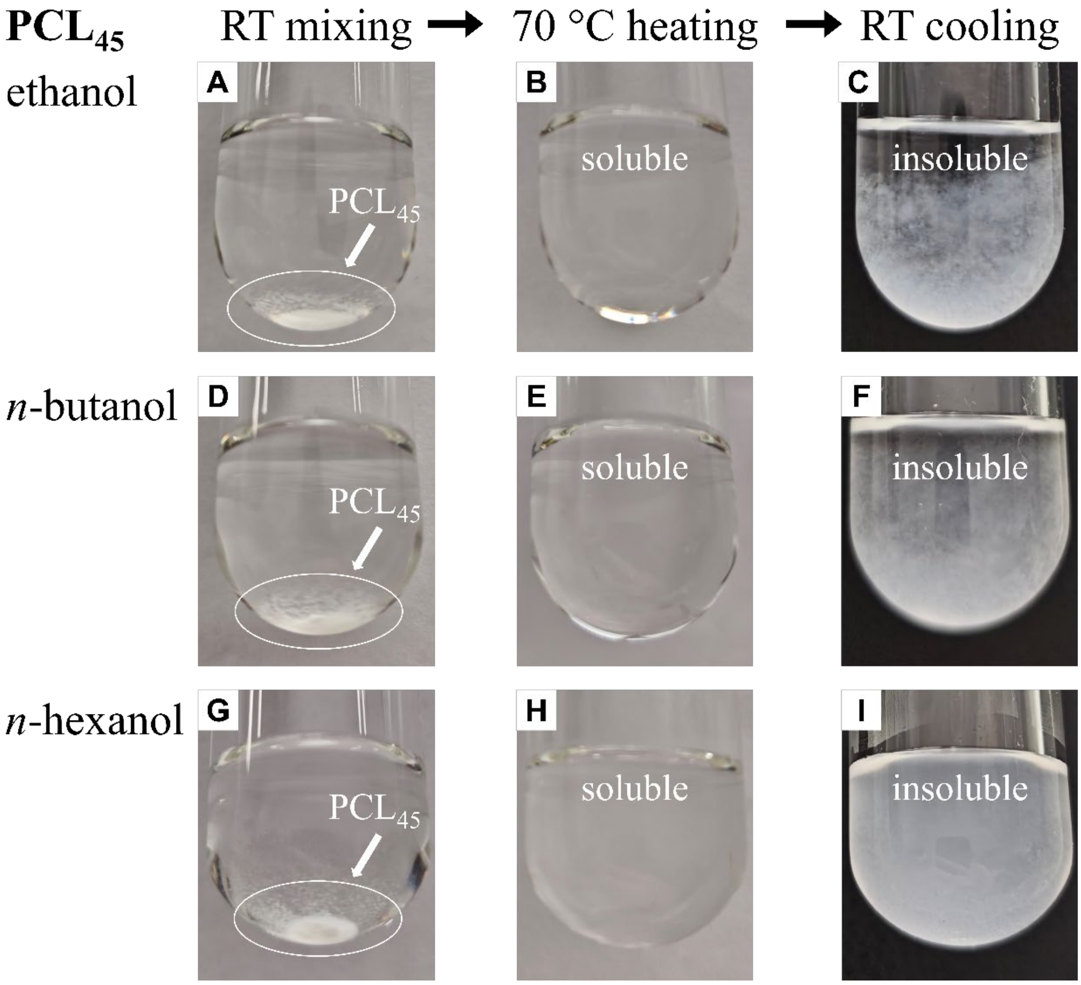

Figure S6. Visual assessment of solubility behavior of PCL_{45} (1 mg/mL). Row order: ethanol, *n*-butanol, *n*-hexanol; Column order: room temperature mixing, 70 °C heating for 1h, room temperature cooling for 12h.

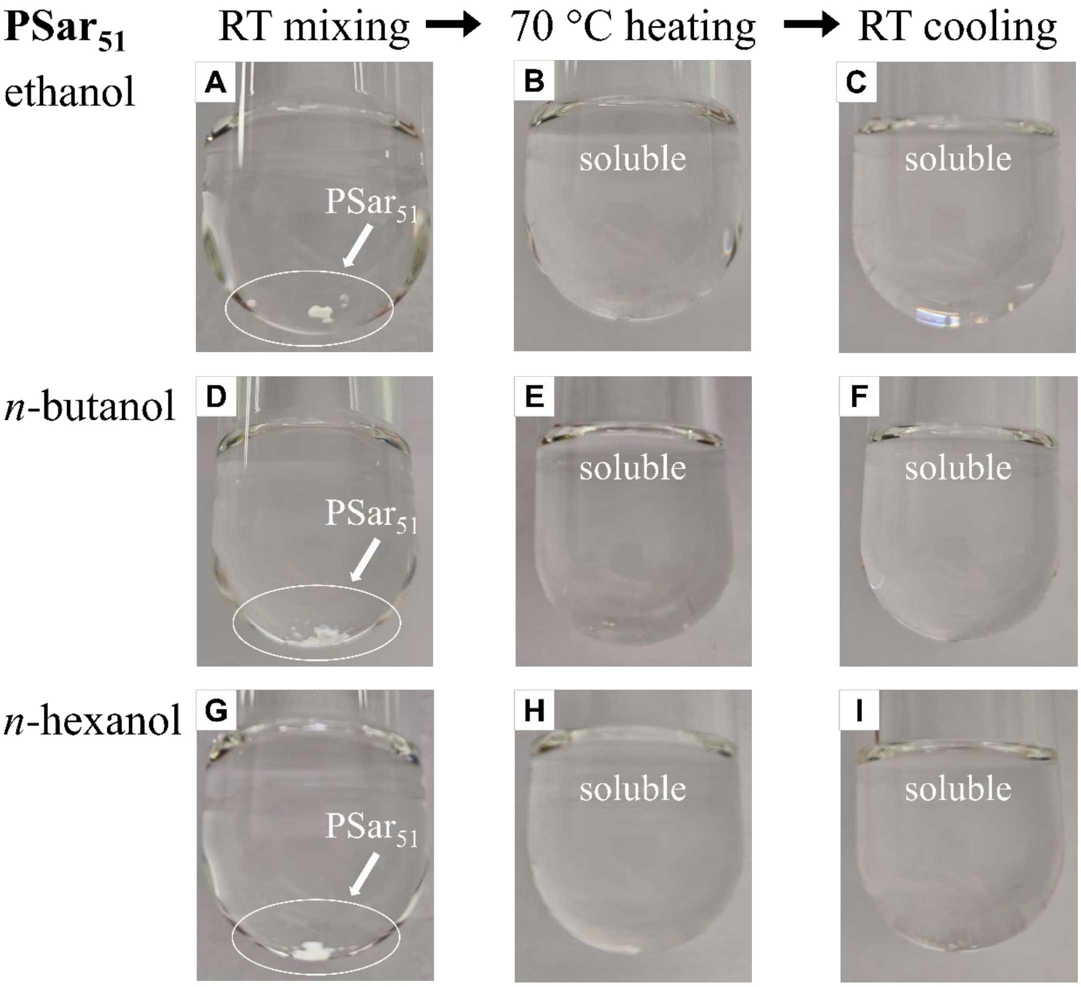

Figure S7. Visual assessment of solubility behavior of $PSar_{51}$ (1 mg/mL). Row order: ethanol, *n*-butanol, *n*-hexanol; Column order: room temperature mixing, 70 °C heating for 1h, room temperature cooling for 12h.

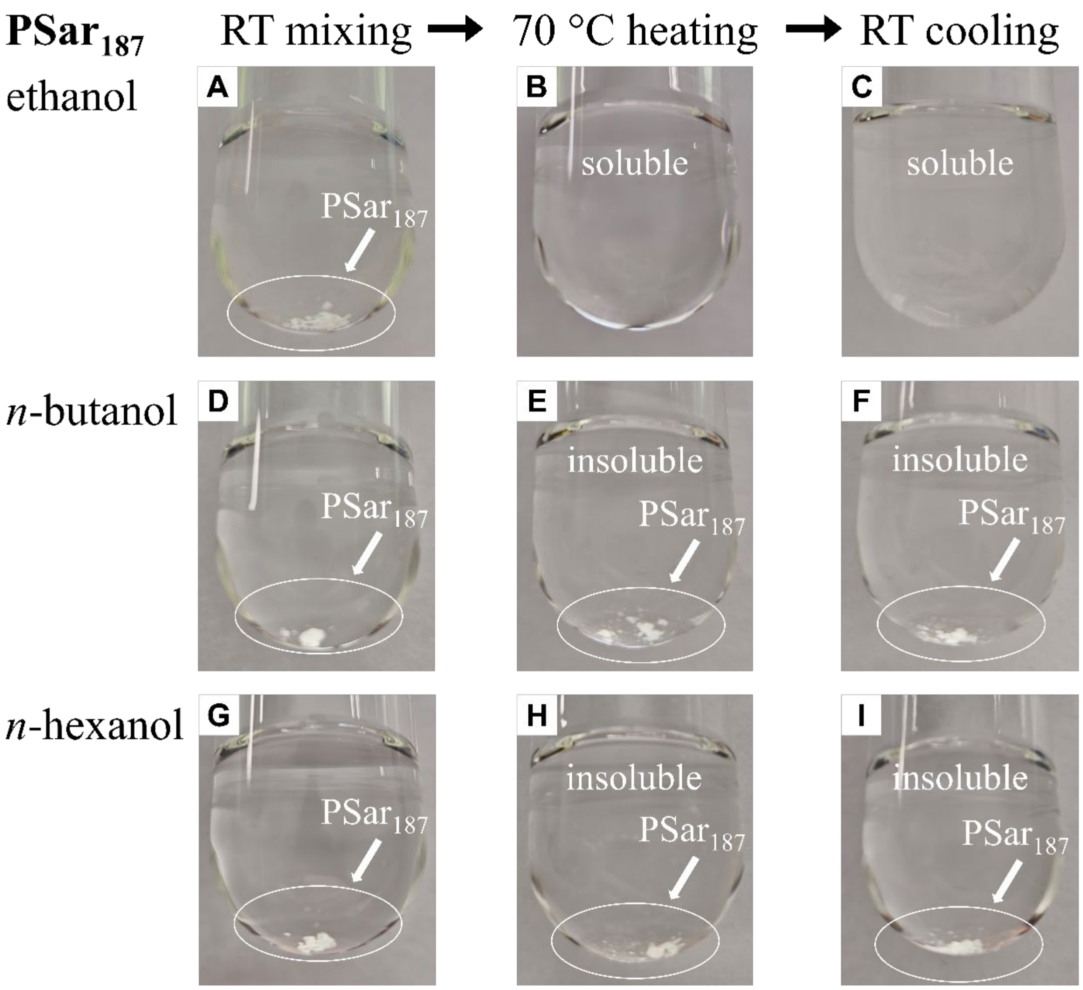

Figure S8. Visual assessment of solubility behavior of $PSar_{187}$ (1 mg/mL). Row order: ethanol, *n*-butanol, *n*-hexanol; Column order: room temperature mixing, 70 °C heating for 1h, room temperature cooling for 12h.
